# National Association of Dental Examiners

**Published:** 1904-10

**Authors:** 


### NATIONAL ASSOCIATION OF DENTAL EXAMINERS.

At the annual meeting held in St. Louis the following officers were elected: President, Thos. J. Barrett, Worcester, Mass.; vice president, of the West, Frank E. Moody, Minneapolis, Minn.; vice president from the South, F. J. Shotwell, Rogersville, Tenn.; vice president from the East, C. Stanley Smith, Cincinnati, O.; secretary and treasurer, C. A. Meeker, Newark, N. J.

---

At a meeting of the Faculties Association held in St. Louis during July, a new rule was adopted in regard to the length of the teaching terms. During the International Dental Congress a great deal of hysterical condemnation of the Faculties was heard because of this "retrogression." It was freely stated that it was a disgrace to American dentistry, for the schools to change from "A four year course of study, to a three year course." Will these gentlemen object if we ask them bluntly, "Why?" And will these immoderate talkers also tell us how it happens to be the concern of the general practitioner who is not connected with any school, whether a dental college takes two or ten years to educate a student? If we reason to a logical deduction, is not this matter of time, exclusively the affair of the contracting parties, the college and the student?

Time! Time! Time! Always Time, as though the length of a term were a true measure of the quality of the education imparted. Is it not conceivable, nay is it not a fact that there are at present existing, schools which teach more in one month than some other schools teach in a full term?

A four-year course, reduced to a three-year course! That is the accusation. The accusers talk glibly of "years," as though a college term covered a twelvemonth. The editor of the *Dental Cosmos,* in his August issue explains that under the so-called "four-year" course the full actual teaching time amounted to twenty-two months, while under the rule adopted at St. Louis, though only occupying "three years," the actual teaching time is twenty-one months. The loss of a month's teaching will seem of small consequence to the average student, in comparison with the year gained. It is probable that no dental graduate ever found himself really competent until he had had at least one full year of actual practice, on his own resources. Compare then the products of the two systems four years after matriculation. One student will have had twenty-two months of college training and lectures for his four years of time, while the other will have had twenty-one months of college training and lectures, plus a full twelve months of actual practice. The first man on commencement day will literally be at his commencement, while the other will already have the foundation of his practice established.

It is not here intended to argue for any lowering of standards, but rather to point out the fallacy of supposing that length of study is a measure of an education. True, four years of high school afford more education than three years of high school. But it is not necessarily true that a four year course in a dental college would produce a better dentist than a three year course, especially if there be but one month's difference in actual attendance.

Of course there must be a minimum time requisite for teaching dentistry, but it would seem that none but those actually engaged in the work of dental education are competent to determine what this minimum time should be. Certainly the average general practitioner is not able to fairly judge; nor is it any of his business.

The general practitioner, however, does have the right to require that those who enter the practice of dentistry, in association and in competition with himself, should at least have definite skill and attainments. In other words, while having no right to criticise the college methods, he does have the right to scrutinize the college product. Moreover, he has seen fit to safeguard his rights, by legislation establishing licensing boards. What then of the boards?

Examining Boards were primarily established for but a single purpose; to inquire into the qualifications of candidates for license to practice dentistry. In other words the definite duty of the examining board is to measure the product of the schools, and to license such only as measure up to the required standard. With the methods of the schools or the durations of their terms they have, or should have no concern.

Yet immediately after the announcement by the Faculties that they will graduate men after twenty-one months of actual teaching, the Examiners Association issues its pronunciamento that graduates must have had "twenty-eight calendar months of college attendance." This is one of those vague rules for which this Association is becoming famous. Does this mean twenty-eight months inclusive or exclusive of holidays and examinations? If the latter, then the Examiners require just seven months more than do the Faculties. The point at issue is that, as with the general practitioner, so with the examining boards. It is none of their business. They have a right to state how much, and not how long a college must teach a student, to render him elligible as an applicant for a license.—*Items of Interest.*

---

Dr. W. O. Talbot, formerly of Biloxi, Miss., has removed to New Orleans and will devote his time exclusively to Orthodontia in the future.

### SHADOW VARIATION IN PORCELAIN INLAY.

By J. L. Newborn, D. D. S., Memphis, Tenn.

(Written for *The American Journal of Dental Science.*)

